# Preparing future general practitioners: the effects of individual, familial, and institutional characteristics

**DOI:** 10.1186/s12909-023-04857-2

**Published:** 2023-11-09

**Authors:** Qiang Su, Dan Hu, Xiaoru Lin, Teng Zhao

**Affiliations:** 1 Zhejiang Academy of Higher Education, Hangzhou Dianzi University, Hangzhou, 310018 China; 2 China Center for Health Development Studies, Peking University, Beijing, 100083 China; 3 School of Marxism, Hangzhou Dianzi University, Hangzhou, 310018 China

**Keywords:** General practitioner, Community-based health service, Medical student, Sex-related difference, China

## Abstract

**Background:**

There is a substantially increasing need for general practitioners (GPs) for future unpredictable pandemic crises, especially at the community-based health services (CBHS) level to protect the vast and varied grassroot-level population in China. Thus, it is crucial to understand the factors that affect Chinese medical students’ GP career choices and commitments to CBHS.

**Methods:**

Leveraging the self-administered data collected across the country, this study conducted logistic regressions with 3,438 medical students. First, descriptive statistics of outcome variables and independent variables were provided. Then, stepwise logistic regression models were built, starting from adding individual characteristics, and then familial and institutional characteristics. Last, post-estimation was conducted to further assess whether there were significant marginal effects.

**Results:**

Results showed that women students were 24% less likely to choose GP careers but were 1.25 times more likely to commit to CBHS than their men peers, holding other individual, familial, and institutional characteristics constant. In addition, students who major in GP-orientated were more likely to choose GP careers and commit to CBHS, respectively, than those who major in clinical medicine. Furthermore, familial characteristics like annual income and mother’s educational level only significantly predicted commitments to CBHS. Notably, sex-related differences in GP career choices and commitments to CBHS – by different regions – were observed.

**Conclusions:**

Understanding the factors that affect medical students’ GP career choices sheds light on how medical education stakeholders can make informed decisions on attracting more medical students to GP-orientated majors, which in turn cultivates more GP professionals to meet the nation’s demand for GPs. In addition, by understanding the factors that influence medical students’ commitment to CBHS, policymakers could make beneficial policies to increase medical students’ motivations to the grassroot-level health institutions, and devote to CBHS as gatekeepers for a large population of residents’ health.

## Introduction

In 2016, the Chinese government issued a health blueprint which emphasized that general practitioner (GP) service is an imperative way to achieve “Healthy China 2030” [1]. In China, GPs are primarily in charge of grassroots and community health centers that provide residents with a full range of medical care to achieve the goal of a hierarchical medical system. Especially in the current post-COVID-19 era, GPs work as the bridge that connects patients and illnesses, reducing the risk of infection and releasing the workload for hospitals.

Since the emergence of the COVID-19 in China, primary healthcare centers have played an unprecedented and critical role in curbing its spread [2]. As such, the needs of GPs have rapidly increased, not only for the COVID-19 but also for future unpredictable pandemic crisis. The government plans to train 35,000 GPs in various ways, particularly focusing on improving community-based health services (CBHS) [3]. Not just in China, there is a shortage of GPs across the world [4]. For example, in the United Kingdom, approximately 30% of all GPs plan to quit direct patient care within five years [5]. In the United States, it is expected to have a shortfall of 7,800 to 32,000 GPs by 2025 [6]. However, young medical students and doctors have been found to have a lower interest in choosing GP careers [7–9]. Thus, it is crucial to find out the factors that would impact medical students – as a GP reserve force – to choose a GP career.

Due to the imbalance in economic development and the diversity in Chinese geography, a large proportion of China’s population is at the grassroot-level, primarily using CBHS [10]. CBHS is mainly delivered by the grassroot-level health care institutions including community health service centers, urban/suburban health centers, and rural clinics [11]. Compared with higher level hospitals, these grassroot-level health institutions have accessed to fewer medical resources but played as the “gatekeepers” of Chinese residents’ health in the first line [12]. However, types and/or professional capacities of medical personnels in CBHS vary largely; there are GPs, doctors with undergraduate medical degrees, assistant physicians, and even unqualified medical personnels in some rural and/or remote areas, which makes governments urgently promote GP trainings to establish a reliable CBHS workforce for the vast and varied grassroot-level population [13]. Given the current landscape and importance of CBHS, it is equally important to understand the determinants of medical students’ commitments to CBHS.

Many have considered China’s medical education system to be one of the most complex in the world [14]. Medical students in China can obtain varying levels of medical degree, allowing them to have distinct medical education pathways and then careers [15]. As the increasing demand for GPs, GP-oriented college majors/specialties have been established since 2010s. Unlike clinical medicine majors that prepare for varying health professionals [16], GP-oriented majors aim to train more future GPs. However, either GP-oriented students or clinical medicine students could have varying career choices due to the perplexing medical education system. These medical students could choose to a health professional career either stick with their college majors or transfer to a specialty after several years training and practices in hospitals. Also, many of them are not willing to be CBHS workers in the grassroot-level health institutions particularly those in rural and/or remote areas [17]. Such contexts present a compelling and critical opportunity to understand what factors may affect medical students’ GP career choices and their commitments to CBHS.

Previous research has revealed a variety of factors as possible influences on the career choices of medical students, including individual, familial, and institutional levels [18–21]. For example, Heiligers [18] found that there were sex differences in medical students’ motivation and career choices. Bittaye et al. [20] included medical students’ familial characteristics such as parental occupation and educational level when examining their choices of specialty. With respect to characteristics of medical schools, Campos-Outcalt and Senf [21] found that they were associated with students’ specialty choices. Focusing on GP career choices, Deutsch et al. [22] found that practice-orientated GP courses offered during undergraduate medical education positively affected students’ GP career choices. In addition, a significant correlation between GP teaching and their future GP careers choices has been found [23]. Building upon this existing literature, we included a richer set of individual, familial, and institutional levels to explore the factors that potentially relate to medical students’ GP career choices.

Notably, research has found that medical students’ attitudes to GP careers may change due to the work environment [24]. It should be noted that there are distinguished differences in the working environment between hospitals and community or rural health services providers in China. GPs based in the community are restricted from prescribing medicines compared to GPs in hospitals, which leads to a lower level of trust between doctors and patients [25]. Moreover, the earnings of those working in hospitals are much higher than those of those working in CBHS. Research has found that medical students’ career choices are associated with their educational debt [26, 27], therefore, it is possible that students are less willing to commit to CBHS.

This study attempts to understand the factors that influence medical students’ GP career choices and commitments to CBHS. Specifically, we mainly address the following two research questions:

1. What individual, familial, and institutional factors will affect Chinese medical students’ GP career choices?
2. What individual, familial, and institutional factors will affect Chinese medical students’ commitments to CBHS?

To our knowledge, the current literature fails to simultaneously address both questions. Using a sample of undergraduate medical students across the country, we conducted logistic regression models to examine the factors that affect students’ GP career choices and commitments to CBHS. It contributes to the global literature on medical students’ pathways to GP careers and CBHS. In practice, these findings are extremely important for China’s medical education stakeholders, which could help to foster more GP professionals and lead them to CBHS, thereby responding to the substantial needs for health services in GP, especially at the CBHS level.

## Materials and methods

### Data and sample

This study leveraged the self-administered data: College General Practice Education (CGPE) to conduct the quantitative analysis. The CGPE suited the present study particularly well because it comprehensively collected medical students’ GP-related information such as GP career choices, CBHS commitments, GP knowledge, and GP internship. Additionally, it collected abundant students’ individual information such as sex, ethnicity, urbanicity, familial information such as household annual income and parental occupations and educations, institutional information such as medical school types, regions, and GP curriculums and teachers.

The CGPE survey was designed by the research team which was supported by the National Social Science Foundation of China. It was distributed to medical students across the country via WJX.CN – a widely used online survey platform in China. The survey period was from June 16 to October 19, 2022. There were two steps in the data collection process. First, we nationally recruited 20 faculties from medical schools using a convenience sampling strategy. Second, these faculties distributed the survey link to their medical students. All the participants were voluntary and were informed that their data would be used confidentially and could not be identified through the research. Validity check was performed before conducting the analysis, yielding a final analytic sample of 3438. Among them, 53.37% were women students, which closely aligned with the female proportion (53.4%) of the whole Chinese university students population from 113 studies [28]. With regards to ethnicity, 89.09% were Han – the socially dominant group in China, and 10.91% were other minorities, such as Zang, Hui, and Man [29]. The corresponding Han proportion was 92.0% in Gao et al. [28], which was also reasonably similar. Therefore, the final analytic sample demonstrated a reasonably unbiased representative of a wider population in terms of sex and ethnicity.

### Measures

#### GP Career choices and CBHS commitments

GP career choices and CBHS commitments were the two dichotomous outcome variables in the present study. Students were asked whether they would choose GP career after graduation, if yes, it was coded as “1”, otherwise, “0”. Similarly, if a student committed to CBHS, it was coded as “1”, otherwise, “0”.

#### Individual characteristics

Except sex and ethnicity, other individual characteristics included urbanicity, college majors, GP familiarity, and GP internship. Urbanicity measured the original places that a student was from, urban, suburban/town, or rural. College majors indicated whether a medical student was majoring in GP-orientated major or clinical medicine major such as internal medicine, pediatrics, neurology, and chirurgery. GP familiarity had two components – familiarity of GP policy and GP knowledge. Familiarity of GP policy measured how familiar a student was with GP-related policies using a five-point Likert scale, with 5 = extremely familiar and 1 = extremely unfamiliar.

To comprehensively measure familiarity of GP knowledge, a series of survey items were consisted of – “are you familiar with differences between general practice and other specialties?”, “are you familiar with general practice or basic health services?”, “are you familiar with the roles of general practitioners in the health service system?”, and “are you familiar with work tasks of general practitioners?”. Reliability test and factor analysis were conducted to assess whether these survey items well measured this generated latent variable [30]. The results showed a high reliability, with Cronbach’s alpha = 0.94 – and high factor loadings ranged from 0.82 to 0.91, meaning 67.24–82.81% of the variances in these items were explained by familiarity of GP knowledge.

Internship has been found to be associated with students’ GP choices [31]. This study also included students’ CBHS internships experience as a dichotomous variable (1 = yes, 0 = no), and their beliefs on current internships using a five-point Likert scale with 5 = extremely useful and 1 = extremely useless.

#### Familial characteristics

Familial characteristics were sorted into four categories. First, since one child policy has substantial effects on the Chinese society [32], we measured students’ sibling status as “1” if a student was from a one-child family, otherwise, “0”. Second, household annual income was categorized into low- (< CNY 50,000), middle- (> CNY50,000 and < CNY200,000), and high- income (> CNY200,000). Third, father’s occupation and education level were included. For simplification, occupations were divided into two categories: government/public institution employees and other jobs; education level were divided into three categories: less than four-year degree, four-year degree, and four-year degree beyond. Fourth, mother’s occupation and education level were coded similarly with these of fathers.

#### Institutional characteristics

Seehusen et al. [33] conducted a systematic review and found that institutional characteristics were likely to affect medical students’ career choices. In this study, we included students’ medical school types, either operated in a comprehensive university (coded as 1) or as an independent school (coded as 0). In addition, based on China’s geographical division, regions of medical schools were sorted into Southwest, North, Eastern, Central, Northwest, and Southern China. Furthermore, GP educational resources such as whether institutions offered GP curriculums and teachers – were likely to affect students’ GP career choices, thereby being included as dichotomous variables (1 = yes, 0 = no).

### Analytic plans

The analysis of this study was conducted by Stata 16. First, descriptive statistics of outcome variables and independent variables were provided. To further understand how students’ GP career choices and CBHS commitments distributed by sex and ethnicity, we conducted Pearson’s chi-squared test. Second, given the outcome variables were binary variables, logistic regressions were performed to examine whether or not the potential student-, family-, and institution-level factors affect medical students’ GP career choices and CHBS commitments. Stepwise logistic regression models were built, starting from adding individual characteristics, and then familial and institutional characteristics. The final logistic regression models could be expressed as follows:

1 $$logit{\left(\frac{{P}_{gp}}{1-{P}_{gp}}\right)}_{i}={\beta }_{0}+{\beta }_{1}{SC}_{i}+{\beta }_{2}{FC}_{i}+{\beta }_{3}{IC}_{i},$$

2 $$logit{\left(\frac{{P}_{cbhs}}{1-{P}_{cbhs}}\right)}_{i}={\beta }_{0}+{\beta }_{1}{SC}_{i}+{\beta }_{2}{FC}_{i}+{\beta }_{3}{IC}_{i},$$

where *gp* = whether student chose a GP career after graduation in Eq. (1); *cbhs* = whether student committed to CBHS after graduation in Eq. (2); ${SC}_{i}$ = a vector of student *i*’s individual characteristics; ${FC}_{i}$ = a vector of student *i*’s familial characteristics; ${SC}_{i}$ = a vector of student *i*’s institutional characteristics.

Noteworthily, we additionally assessed whether these individual, familial, and institutional characteristics were too highly correlated with each other. Specifically, we checked the variance inflation factors (VIFs) for multicollinearity before conducting logistic regressions. The results showed that the largest VIF for institutions located in Central China was 2.40, suggesting that multicollinearity may not need to be taken into account.

Last, post-estimation was conducted to further assess whether there were significant marginal effects. In other words, since medical students’ GP choices and CBHS commitments were likely to be associated with their sex, we were particularly interested in whether these relationships varied by regions, as China’s economic growth has been unbalanced among regions.

## Results

### Descriptive statistics

Table 1 reports the distribution of medical students’ GP career choices and CBHS commitments by sex and ethnicity. Chi-square tests were included to assess the significant differences in the distribution of GP career/Non-GP career choices and CBHS/non-CBHS commitments among students. First, with respect to GP career choices, we observed significant differences by sex (χ2 = 19.23, *p* < 0.001) and insignificant differences by ethnicity (χ2 = 2.48, *p* > 0.05). Specifically, we found that 67.74% of women would like to choose GP careers as compared to 74.55% of men.

**Table 1:** Outcome variable distribution by sex and ethnicity

Predicting General Practitioner Careers				
	Sex (χ2 = 19.23, *p* < 0.001)		Ethnicity (χ2 = 2.48, *p* > 0.05)	
	Men	Women	Han	Others
GP career	1,195	1,243	2,159	279
	74.55%	67.74%	70.49%	74.40%
Non-GP career	408	592	904	96
	25.45%	32.26%	29.51%	25.60%
Total	1,603	1,835	3,063	375
**Predicting Community-based Health Service Commitments**				
	**Sex (χ2 = 7.89**, ***p*** **< 0.01)**		**Ethnicity (χ2 = 7.70**, ***p*** **< 0.01)**	
	Men	Women	Han	Others
CBHS	949	1,172	1,865	256
	59.20%	63.87%	60.89%	68.27%
Non-CBHS	654	663	1,198	119
	40.80%	36.13%	39.11%	31.73%
Total	1,603	1,835	3,063	375

Then, we turned to focus on the disparities in CBHS vs. non-CBHS commitments, which we found a different pattern – varied significantly by sex (χ2 = 7.89, *p* < 0.01) and ethnicity (χ2 = 7.70, *p* < 0.01). We found that 63.87% of women would like to commit to CBHS, as compared to 59.2% of men. With respect to ethnicity, there was an approximate 7.5% difference (between Han students at 60.89% and other ethnicities at 68.27%) in CBHS commitments.

Table 2 reports the descriptive statistics for independent variables. For dichotomous variables, percentage results were reported in the main text instead of mean results in Table 2. Concerning students’ individual level characteristics, 26% of students were from urban areas, while 22% were from suburban/town and 52% were from rural areas. The majority of students (77%) were majoring in clinical medicine. With respect to the familiarity of GP, the mean of students’ familiarity of GP policy and knowledge was 2.91 and 3.31, respectively, around “moderately familiar”. For GP internship, 33% of students had a CBHS internship and the mean of beliefs on current internship was at 2.45, which ranged between “neutral” and “useless”.

**Table 2:** Descriptive statistics for independent variables

Variables	N	Mean	SD	Min	Max
**Individual Level**					
***Sex***					
Women	1,835	0.53	0.50	0	1
Men	1,603	0.47	0.50	0	1
***Ethnicity***					
Han	3,063	0.89	0.31	0	1
Others	375	0.11	0.31	0	1
***Urbanicity***					
Urban	892	0.26	0.44	0	1
Suburban/town	773	0.22	0.42	0	1
Rural	1,773	0.52	0.50	0	1
***Majors***					
GP orientation	806	0.23	0.42	0	1
Clinical medicine	2,632	0.77	0.42	0	1
***GP Familiarity***					
GP policy	3,438	2.92	0.85	1	5
GP knowledge	3,438	3.31	0.92	1	5
***GP Internship***					
CBHS internship (Yes)	1,138	0.33	0.47	0	1
Beliefs on internship	3,438	2.45	0.81	1	5
**Familial Level**					
***One Child Policy***					
Only child (Yes)	1,109	0.32	0.47	0	1
***Household Annual Income***					
Low-income	1,902	0.55	0.50	0	1
Mid-income	1,325	0.39	0.49	0	1
High-income	211	0.06	0.24	0	1
***Father’s Job & Education Level***					
Governments/public institutions	703	0.20	0.40	0	1
Other jobs	2,735	0.80	0.40	0	1
< 4-years degree	2,657	0.77	0.42	0	1
4-years degree	717	0.21	0.41	0	1
> 4-years degree	64	0.02	0.14	0	1
***Mother’s Job & Education Level***					
Governments/public institutions	663	0.19	0.39	0	1
Other jobs	2,775	0.81	0.39	0	1
< 4-years degree	2,841	0.83	0.38	0	1
4-years degree	558	0.16	0.37	0	1
> 4-years degree	39	0.01	0.11	0	1
**Institutional Level**					
***Medical School Type***					
Comprehensive university	1,871	0.54	0.50	0	1
Independent medical school	1,567	0.46	0.50	0	1
***Region***					
Southwest	749	0.22	0.41	0	1
North	29	0.01	0.09	0	1
Eastern	1,045	0.30	0.46	0	1
Central	981	0.29	0.45	0	1
Northwest	425	0.12	0.33	0	1
Southern	209	0.06	0.24	0	1
***GP Education***					
GP curriculums (Yes)	2,869	0.83	0.37	0	1
GP teachers (Yes)	2,646	0.77	0.42	0	1

*Note*. Total N = 3,438. SD = standard deviation. For consistency, means were reported for all dichotomous and continuous variables

With respect to familial characteristics, 32% of students were from only-child family, and 55% were from low-income families. Governments/public institution employees comprised about 1/5 of father’s and mother’s occupations, with 20% and 19%, respectively. Notably, majority of fathers and mothers had a less than four-year college degree, with 77% and 83%, respectively.

Turning to institutional characteristics, 54% of students were studying at a medical school located in a comprehensive university, while 46% were studying at an independent medical school. Students from medical schools located in Eastern China consisted of the largest share, with a 30%. In addition, 83% and 77% of medical schools offered specific GP curriculums and teachers, respectively.

### Predicting medical students’ GP career choices

Table 3 reports the results from a series of logistic regression models in predicting medical students’ GP career choices. For easier interpretation, odds ratios (OR) were reported. Model 1 shows that women were 25% less likely than men to choose a GP career, holding other variables constant (omitted this for simplification when reporting other regression results below). In addition, students from GP-orientated majors were 4.53 times more likely to choose a GP career than those from clinical medicine majors. Interestingly, students who were more familiar with GP policy (OR = 0.70, *p* < 0.001) and knowledge (OR = 0.82, *p* < 0.01) were less likely to choose GP careers. Notably, students who had CBHS internships were 1.97 times more likely to choose GP careers. Model 2 introduces familial characteristics, as additional predictors that may affect students’ GP career choices. However, no additional significant relationships were observed after controlling these familial characteristics.

**Table 3:** Predicting general practitioner career choices among medical students

Variables	Model 1		Model 2		Model 3	
	OR	SE	OR	SE	OR	SE
Sex						
Women	0.75***	0.06	0.75***	0.06	0.76***	0.06
Ethnicity						
Han	0.90	0.12	0.92	0.12	0.84	0.13
Urbanicity (Ref: rural)						
Urban	0.87	0.08	0.98	0.12	1.02	0.12
Suburban/town	0.96	0.10	1.03	0.11	1.06	0.12
Major (Ref: clinical medicine)						
GP orientation	4.53***	0.60	4.53***	0.61	4.04***	0.55
GP Familiarity						
GP policy	0.70***	0.04	0.70***	0.04	0.71***	0.04
GP knowledge	0.82**	0.05	0.82**	0.05	0.85*	0.06
GP Internship						
CBHS internship	1.97***	0.18	1.96***	0.18	1.99	0.19
Beliefs on internship	0.95	0.05	0.94	0.05	1.01	0.05
Only child			0.89	0.08	0.93	0.09
Household Annual Income (Ref: low-income)						
Mid-income			0.95	0.09	0.89	0.08
High-income			0.84	0.15	0.79	0.14
Father’s Job & Education Level						
Governments/public institutions			0.97	0.13	0.96	0.13
4-years degree (Ref: <4-years degree)			1.17	0.15	1.16	0.15
> 4-years degree			1.43	0.48	1.43	0.48
Mother’s Job & Education Level						
Governments/public institutions			0.83	0.11	0.79	0.11
4-years degree (Ref: <4-years degree)			0.94	0.13	0.94	0.13
> 4-years degree			1.01	0.41	0.98	0.40
Medical School Type (Ref: independent medical school)						
Comprehensive university					1.44***	0.15
Region (Ref: Southwest)						
North					0.46	0.20
Eastern					1.37**	0.16
Central					0.59***	0.08
Northwest					0.84	0.14
Southern					0.91	0.17
GP curriculums					1.19	0.16
GP teachers					1.24	0.15
Constant	14.44***	5.85	14.85***	6.04	7.93***	3.42

*Note*. * *p* < 0.05, ** *p* < 0.01, *** *p* < 0.001. N = 3,438. OR = odds ratio, SE = standard error

Model 3 further introduces institutional characteristics. The results showed that sex, GP-orientated major, and the familiarity of GP policy and knowledge remained significantly in predicting students’ GP career choices. Additionally, we observed that students from medical schools in comprehensive universities were 1.44 times more likely to choose GP careers. Notably, comparing to Southwest region, students from Eastern region were more likely to choose GP careers (OR = 1.37, *p* < 0.01), while those from Central region had a lower probability (OR = 0.59, *p* < 0.001).

### Predicting medical students’ CBHS commitments

Table 4 reports the results of factors in predicting medical students’ CBHS commitments. Model 4 shows that women were 1.24 times more likely than men to commit to CBHS. In addition, students from urban areas were 36% less likely to commit to CBHS than those from rural areas. GP-orientated major was still a strong predictor for CBHS commitments (OR = 2.38, *p* < 0.001). Similar to the analytic process in predicting GP careers above, Model 5 shows that students’ beliefs on current internship (OR = 0.77, *p* < 0.001) and mothers with four-year college degree and above (OR = 0.33, *p* < 0.01) – significantly and negatively predicted their CBHS commitments, after controlling familial characteristics.

**Table 4:** Predicting community-based health services commitments among medical students

Variables	Model 4		Model 5		Model 6	
	OR	SE	OR	SE	OR	SE
Sex						
Women	1.24**	0.09	1.24**	0.09	1.25**	0.09
Ethnicity						
Han	0.85	0.10	0.86	0.11	0.72*	0.10
Urbanicity (Ref: rural)						
Urban	0.64***	0.06	0.83	0.09	0.85	0.09
Suburban/town	0.89	0.08	1.04	0.11	1.07	0.11
Major (Ref: clinical medicine)						
GP orientation	2.38***	0.24	2.33***	0.23	2.35***	0.25
GP Familiarity						
GP policy	0.90	0.05	0.89*	0.05	0.90	0.05
GP knowledge	1.02	0.06	1.04	0.06	1.10	0.07
GP Internship						
CBHS internship	1.15	0.09	1.13	0.09	1.20*	0.10
Beliefs on internship	0.77	0.04	0.77***	0.04	0.80***	0.04
Only child			1.02	0.09	1.01	0.09
Household Annual Income (Ref: low-income)						
Mid-income			1.03	0.09	0.97	0.08
High-income			0.75	0.13	0.69*	0.12
Father’s Job & Education Level						
Governments/public institutions			0.88	0.11	0.86	0.11
4-years degree (Ref: <4-years degree)			0.90	0.10	0.90	0.10
> 4-years degree			1.72	0.55	1.72	0.55
Mother’s Job & Education Level						
Governments/public institutions			0.83	0.10	0.80	0.10
4-years degree (Ref: <4-years degree)			0.80	0.10	0.81	0.10
> 4-years degree			0.33**	0.14	0.32**	0.13
Medical School Type (Ref: independent medical school)						
Comprehensive university					1.39***	0.13
Region (Ref: Southwest)						
North					0.28***	0.11
Eastern					0.83	0.09
Central					0.47***	0.06
Northwest					0.43***	0.06
Southern					0.82	0.14
GP curriculums					1.03	0.13
GP teachers					1.29*	0.14
Constant	3.91***	1.44	3.96***	1.47	3.52**	1.40

*Note*. * *p* < 0.05, ** *p* < 0.01, *** *p* < 0.001. N = 3,438. OR = odds ratio, SE = standard error

Including all individual, familial, and institutional characteristics, Model 6 displays that Han students were 28% less likely than other minority students to commit to CBHS. We also found that students who had CBHS internships were 1.2 times more likely to commit to CBHS than those who had not. In addition, students from high-income families were 31% less likely to commit to CBHS than those from low-income families. Regional differences were also observed. Comparing to Southwest region, North, Central, and Northwest regions were 72%, 53%, and 57% less likely to make CBHS commitments, respectively.

### Marginal effects of sex on GP careers and CBHS commitments by regions

**Fig. 1:**
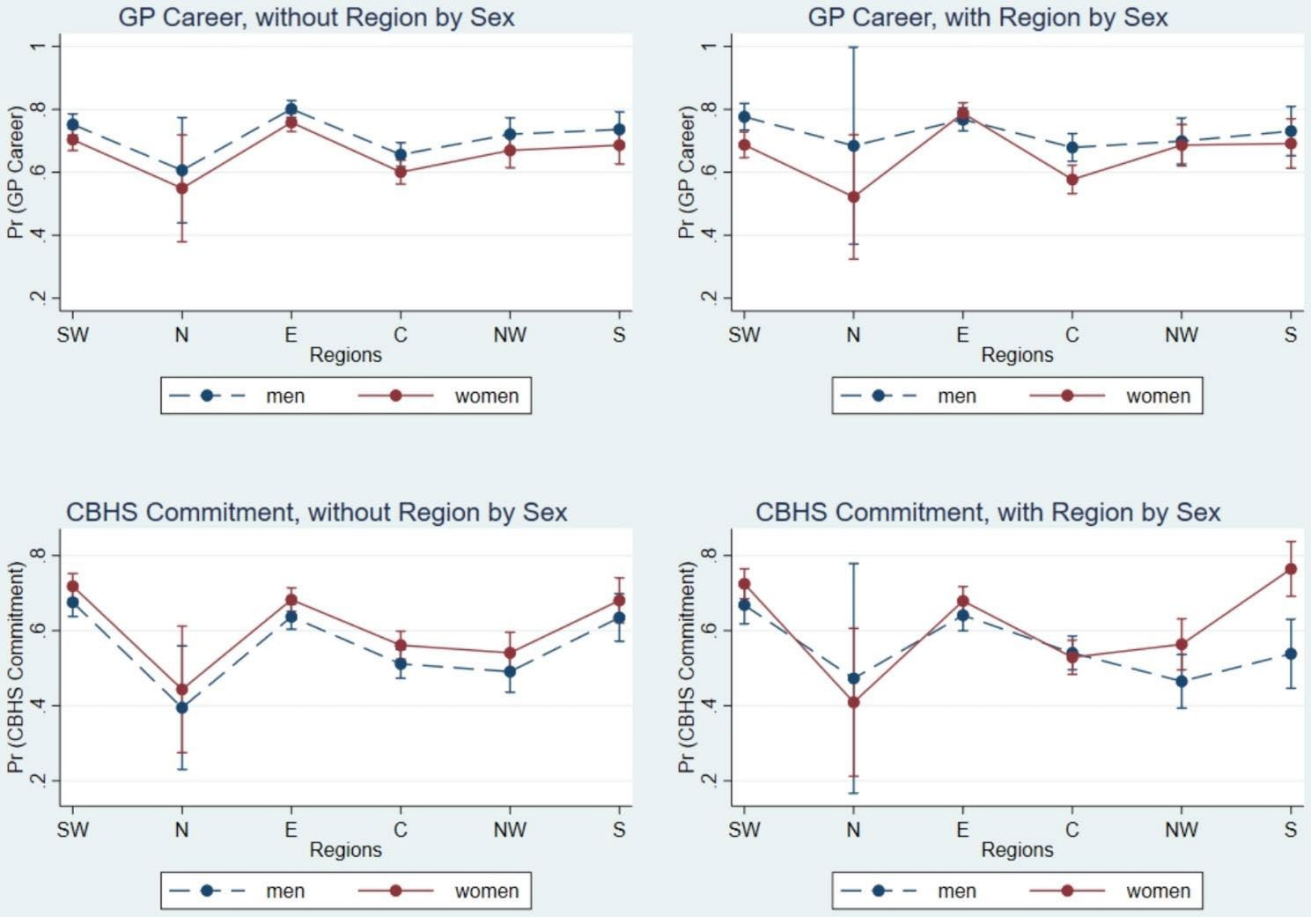
Predictive margins of GP career and CBHS commitment, with and without region by sex. *Note*. SW = Southwest, N = North, E = Eastern, C = Central, NW = Northwest, S = Southern

From above logistic regression results, we observed significant relationships between sex and regions and GP career choices, and between sex and regions and CBHS commitments. To further explore whether the relationships between sex and GP career choices and CBHS commitments were varied by regions, we added interaction terms – sex by regions in our final preferred Model 3 and Model 6, which included all individual, familial, and institutional characteristics. Figure 1 displays that the lines of women and men were not visually parallel, after adding the interaction terms, indicating that these relationships did vary by regions.

**Fig. 2:**
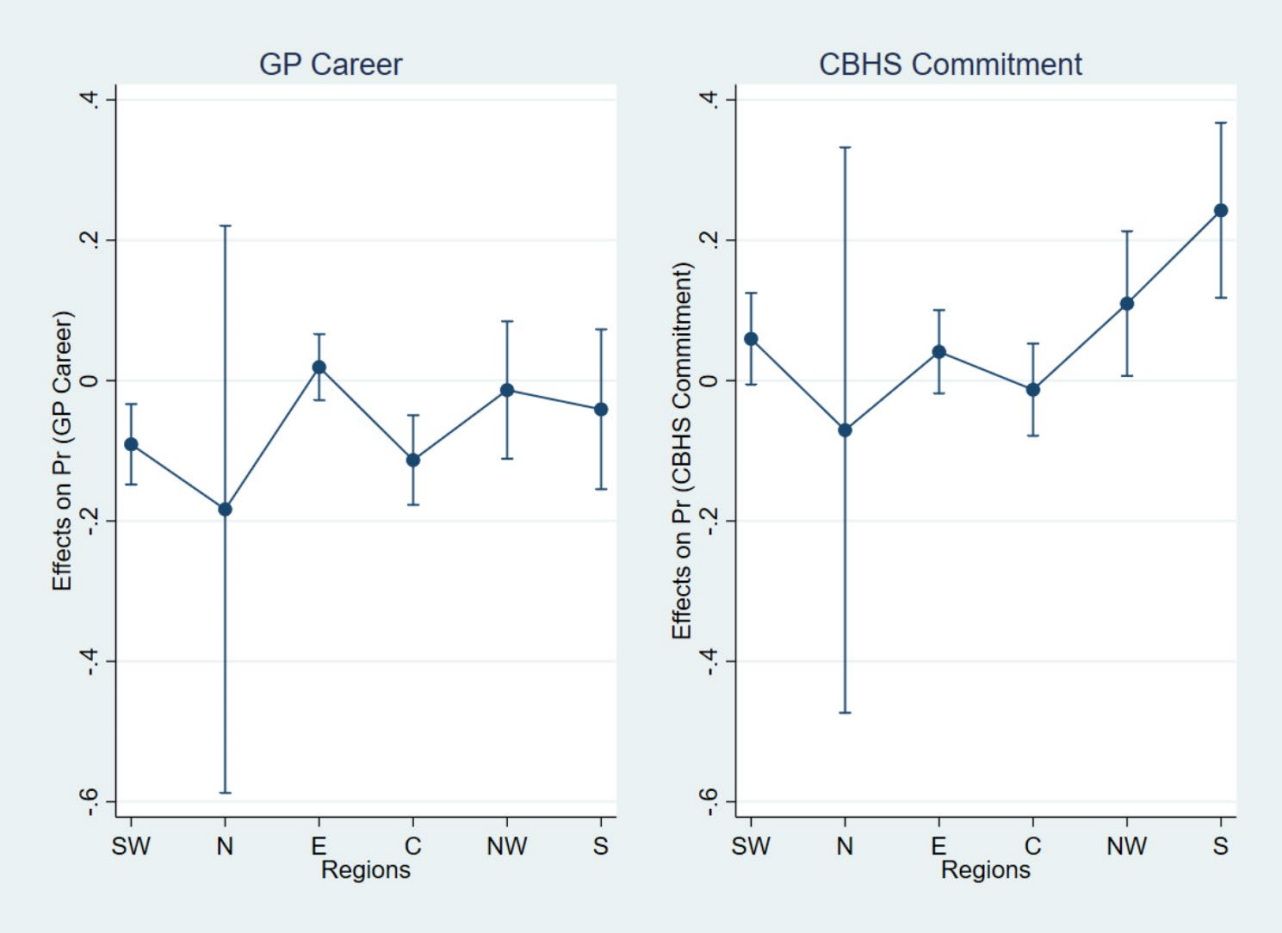
Marginal effects of sex on GP career and CBHS commitment by regions. *Note*. SW = Southwest, N = North, E = Eastern, C = Central, NW = Northwest, S = Southern

Table 5 reports the results of marginal effects of sex on GP careers and CBHS commitments by regions, while Fig. 2 visually displays these marginal effects. Results showed that in Southwest region, women were 9% less likely than men to choose GP careers, while this value was 11% in Central region. With respect to CBHS commitments, women were 11% and 24% more likely than their counterparts to commit to CBHS in Northwest and Southern regions, respectively. No significant differences between sex groups were found in other regions.

**Table 5:** Marginal effects of sex on GP careers and CBHS commitments by regions

Interaction terms	GP careers		CBHS commitments	
	ME	SE	ME	SE
Women*Southwest	-0.09**	0.03	0.06	0.03
Women*North	-0.18	0.21	-0.07	0.21
Women*Eastern	0.02	0.02	0.04	0.03
Women*Central	-0.11***	0.03	-0.01	0.03
Women*Northwest	-0.01	0.05	0.11*	0.05
Women*Southern	-0.04	0.06	0.24***	0.06

*Note.* * *p* < 0.05, ** *p* < 0.01, *** *p* < 0.001. N = 3,438. ME = marginal effect, SE = standard error

## Discussion

This study investigates the factors that influence medical students’ GP career choices as well as commitments to CBHS. Our results show that individual factors like students’ sex, GP-orientated majors, and institutional factors like regions significantly predicted both GP career choices and commitments to CBHS. Familial characteristics like annual income and mother’s educational level only significantly predicted commitments to CBHS.

Specifically, our results show that women students were less likely to choose GP careers, which is inconsistent with Lefevre et al. [34], which found that 76.3% of women students would choose GP careers in France. It is possible that the inconsistency is due to different GPs’ workloads in different countries. China has one of the largest populations of patients in the world, which requires GPs to work out-of-hours regularly. Past studies like French et al. [35] have found that in Scotland, men GPs worked more hours and were more likely to work after hours than their women peers. Thus, Chinese women students may not choose GP careers in order to avoid the extreme workload of GPs. In addition, we find that women students were more willing to commit to CBHS than their male peers. This is consistent with Chuenkongkaew et al. [36], which surveyed medical students from five Asian countries and found that female medical students were more likely to work in rural areas than their male peers. This is probably because those who commit to CBHS require high empathy, and women in general are more empathetic due to maternal instinct [37]. Another reason may be that in China, CBHS usually does not need to work after hours, however, primary hospitals do.

Unsurprisingly, our results reveal that students who majored in GP-oriented majors were more likely to choose GP careers and commit to CBHS than those who majored in clinical medicine. Many clinical medicine students believe that GP careers have lower salaries and are not valued by professionals [38]. Therefore, they prefer to stick with their major. However, students in GP-oriented majors may put family first and pay more attention to the flexibility of working hours [39], thereby choosing GP careers to have a better work-life balance. With respect to committing to CBHS, our findings are supported by Gill et al. [40], which demonstrated that students in GP-oriented majors had a preference for working in a rural community and placed more emphasis on continuity of care.

Interestingly, our results show that students who are knowledgeable about GP-related policy and knowledge do not choose GPs, which may suggest that GP-related policy and knowledge mastered by medical students did not meet their expectations. The existing research has found that curriculum, internships, and the power of role models play an imperative role in the choice of GP careers [22, 41]. However, current medical education in China is plagued by issues such as one-sided teaching of the general practice curriculum, short-term rural-oriented internships, or teachers’ failure to act as positive role models, which will cause students’ unsatisfaction with GP careers. Furthermore, GP-related policies have unattractive salary provisions [42], which are much lower than those of specialties. These help explain why students who are more knowledgeable about GP, on the contrary, are less likely to choose GP careers.

With respect to familial characteristics, we find that students from high-income families were less likely to commit to CBHS. According to Nicholson et al. [19, 36], medical students’ pre-med choices were also influenced by their families. Thus, it is possible that high-income families tend to invest more in their children’s education and expect them to have more decent jobs. However, GP careers have a low social status in China which may decrease high-income students’ interests. This new finding may have implications on establishing a GP-welcoming social environment to improve GPs’ social status. In addition, we also found that students whose mothers had more than a four-year college degree had less probability of committing to CBHS. This is consistent with Liu et al. [36], which surveyed 232 medical students and found that medical students’ attitude toward remaining in rural areas was strongly associated with mothers’ education level at postsecondary or above.

With respect to institutional characteristics, we find there are regional differences in GP career choices and commitments to CBHS. Specifically, students in the Eastern region were more likely than Southwest region students to pursue a GP career. Eastern region in China is generally regarded as the economically high-developed region [43]. Research has found that general practitioners with low salaries have lower job satisfaction [44]. It is reasonable that GPs in economically high-developed regions have higher salaries than economically less-developed regions like Southwest, and have higher job satisfaction, which in turn, influences medical students’ GP careers. To our knowledge, limited existing literature has explored regional differences in medical students’ intentions to GP careers, meanwhile the present study contributes to this research gap.

In addition, students in the Southwest region––where has a higher rural density––were more willing to commit to CBHS. This is consistent with Zhang et al. [45], which was conducted among 2,714 medical students from three medical schools, suggesting that students from rural areas were more likely to choose to work in communities than those from urban areas. Similarly in Australia, after surveying the employment locations of medical graduates at 12 universities, McGirr et al. [46] demonstrated that medical students with rural backgrounds were 3.1 times more likely to practice medicine in rural areas than medical students with metropolitan backgrounds.

Moreover, we found that sex plays an important role in regional differences in GP career choices and commitments to CBHS. Women in Chinese traditional culture are often seen as “homemakers” whose primary role is to take care of the family. These prejudices have lasted over time, especially in the areas of gender inequity, where most people still believe that it is a woman’s job to take care of the family [47], thereby avoiding jobs with high workloads and choosing to work close to home [48]. This could help to partially explain our finding that women students in Southwest region are less likely to choose GP careers, and women students in Southern region were more likely to commit to CBHS, as both regions were known for gender inequality. However, the reasons of insignificant results of Southwest women students’ GP career choices and Southern women students’ commitments to CBHS, remain unclear, and could not be explored by the present study.

Despite comprehensively design the present study, three limitations should be noted. First, although the study included individual, familial, and institutional characteristics, other students’ psychological variables could also be generated. Studies like Lent et al. [49] conducted structural equation models to better understand how psychological variables predicted students’ career choices. In addition, specific majors could be collected. Thus, future research could redesign the study and add new insights to the influential factors that affect medical students’ GP career choices and commitments to CBHS, by specific majors. Second, the present study could not conclude a causal relationship. Causal inferences are important to evidence-based administrations [50]. Future research could conduct quasi-experiment designs such as propensity score matching to reduce selection biases. However, it is still valuable for stakeholders to understand what individual, familial, and institutional characteristics impact students’ GP career choices and commitments to CBHS. Third, the measure of GP career choices was not the declared GP careers, instead, we used medical students’ willingness to choose GP careers. However, this could still inform stakeholders with practical implications on the cultivation of GPs. Future research could survey GPs rather than medical students to more explicitly identify these potential factors.

## Conclusions

Understanding the factors that affect medical students’ GP career choices is an important alternative to respond to the increasing needs for GPs in China. Additionally, understanding the factors that affect students’ commitments to CBHS can contribute to establish a reliable CBHS workforce that can provide first line protection for the numerous and diverse grassroot-level population. This study sought to explore these influential factors. The results indicated that individual factors like students’ sex, GP-orientated majors, and institutional factors like regions significantly predicted both GP career choices and commitments to CBHS. Familial characteristics like annual income and mother’s educational level only significantly predicted commitments to CBHS. It should be noted that GP-orientated majors have been established since 2010s to cultivate more GPs, and our results suggested that GP-orientated majors do function well in such a role. In addition, by understanding these influential factors, medical education stakeholders can make informed decisions on attracting more medical students to GP-orientated majors, which in turn cultivates more GP professionals to meet the nation’s demand for GPs. Furthermore, by understanding the factors that influence medical students’ commitment to CBHS, policymakers could make beneficial policies to increase medical students’ motivations to the grassroot-level health institutions, and devote to CBHS as gatekeepers for a large population of residents’ health.

## Data Availability

The datasets used and analyzed during the present study are not publicly available due to the protection of participants privacy, but are available from the corresponding author upon reasonable request.

## Abbreviations

CBHS: Community-based health services
CNY: Chinese Yuan
GP: General practitioners
OR: Odds ratios
VIF: Variance inflation factors
